# Rho Signaling in Synaptic Plasticity, Memory, and Brain Disorders

**DOI:** 10.3389/fcell.2021.729076

**Published:** 2021-10-04

**Authors:** Haorui Zhang, Youssif Ben Zablah, Haiwang Zhang, Zhengping Jia

**Affiliations:** ^1^Program in Neurosciences and Mental Health, The Hospital for Sick Children, Peter Gilgan Centre for Research and Learning, Toronto, ON, Canada; ^2^Department of Physiology, Temerty Faculty of Medicine, University of Toronto, Toronto, ON, Canada

**Keywords:** Rho GTPases, long-term potentiation, dendritic spine, long-term depression, brain disorders

## Abstract

Memory impairments are associated with many brain disorders such as autism, Alzheimer’s disease, and depression. Forming memories involves modifications of synaptic transmission and spine morphology. The Rho family small GTPases are key regulators of synaptic plasticity by affecting various downstream molecules to remodel the actin cytoskeleton. In this paper, we will review recent studies on the roles of Rho proteins in the regulation of hippocampal long-term potentiation (LTP) and long-term depression (LTD), the most extensively studied forms of synaptic plasticity widely regarded as cellular mechanisms for learning and memory. We will also discuss the involvement of Rho signaling in spine morphology, the structural basis of synaptic plasticity and memory formation. Finally, we will review the association between brain disorders and abnormalities of Rho function. It is expected that studying Rho signaling at the synapse will contribute to the understanding of how memory is formed and disrupted in diseases.

## Introduction

The synapse is a highly specialized structure connecting two cells and it serves as the main site where neurons communicate and transmit signals between each other. Synaptic connections allow the formation and function of neuronal circuits that underlie our emotions, behaviors and memories. Synaptic structure was firstly described by Gray (1959), and it consists of vesicle-bearing pre-synaptic terminals that originate from axons and the post-synaptic component found on the cell body, dendrites or a dendritic spines. Many synaptic proteins such as neuroligins and neurexins were identified to maintain the connectivity and precise alignment of pre- and post-synaptic membrane that shapes the amplitude and reliability of neurotransmission (Missler et al., 2012; Tang et al., 2016; Hass et al., 2018). In the mammalian central nervous system, dendritic spines are the primary sites of excitatory inputs (Bourne and Harris, 2008). Morphological changes of dendritic spines in the pre-existing synapses, as well as the *de novo* growth or retraction of dendritic spines, are closely linked to the functional and structural plasticity of the synapse, which is widely believed to be the basis of memory and cognition (Yuste and Bonhoeffer, 2001; Yang et al., 2008; Holtmaat and Svoboda, 2009; Kasai et al., 2010; Chidambaram et al., 2019).

Dendritic spines are small protrusions that emerge on dendritic processes, and they occur at a density of 1–10 spines/μm (Sorra and Harris, 2000). Dendritic spines are heterogenous in size and shape and can be classified into several categories based on their morphological differences. Typically, a dendritic spine consists of three basic compartments: (1) a delta-shaped base which contains branched actin filaments residing on the microtubule network in the dendrites, (2) a constricted neck in the middle which contains branched and linear longitudinal actin filaments, and (3) a bulbous head contacting the axon, which usually contains a dense network of short cross-linked branched actin filament (Hotulainen and Hoogenraad, 2010). Based on the size of spine heads and the length of spine necks, dendritic spines can be subdivided into mushroom, thin, branched, and stubby spines (Peters and Kaiserman-Abramof, 1970; Harris et al., 1992). A mushroom spine contains a narrow neck with a large head. Since the synaptic connections formed on the mushroom spine can usually last for a long time, it is also considered to be a mature “memory spine,” where the long-term memory is stored (Bourne and Harris, 2007). Thin spines have a similar structure as mushroom spines, except that the spine head is smaller. Thin spines are more dynamic in their structure, having the capacity to transform into mushroom spines and therefore, are considered to be “learning spines” (Bourne and Harris, 2007). Stubby spines typically have an indiscernible neck and mainly exist during the early stage of postnatal development (Fiala et al., 1998). Branched or cup-shaped spines have multiple heads that originate from a single neck. It is important to note that this classification of spines may underrate the morphological heterogeneity of spines because there is a continuum of spine sizes and shapes that are also highly dynamic (Fiala et al., 1998; Parnass et al., 2000; Alvarez and Sabatini, 2007; Pchitskaya and Bezprozvanny, 2020).

A complex actin network that is associated with various actin regulators, including the Arp2/3 complex and other actin-binding proteins, constitutes the main component of spine cytoskeletal architecture, and therefore, the formation, maturation and plasticity of the spine are highly dependent on the remodeling of the actin cytoskeleton (Cingolani and Goda, 2008; Hotulainen and Hoogenraad, 2010; Korobova and Svitkina, 2010). The Rho family small GTPases are the central mediators of actin reorganization in many cell types, including neurons. They are molecular switches cycling between the active GTP-bound and inactive GDP-bound form, and their activities are tightly regulated by guanine nucleotide-exchange factors (GEFs) and GTPase-activating proteins (GAPs) (Govek et al., 2005; Vega and Ridley, 2007). Among Rho family members, RhoA, Rac1, and Cdc42 are the most extensively studied in the brain and are the focus of this review. RhoA, Rac1, and Cdc42 were initially found to be required in the growth factor-induced formation of focal adhesions and actin stress fibers (contractile actin and myosin filaments), membrane ruffles/lamellipodia (meshwork of newly polymerized actin filaments), and filopodia (actin-rich and finger-like membrane extension structures) in fibroblast cells (Ridley et al., 1992; Ridley and Hall, 1992; Nobes and Hall, 1995). Subsequent studies have shown that these Rho GTPases are crucial in the regulation of various aspects of cell morphology such as cell polarity and shape as well as several cellular processes such as exocytosis, endocytosis and proliferation, in a wide variety of mammalian cell types, including neurons (Etienne-Manneville and Hall, 2002; Govek et al., 2005).

## Rho GTPases in Spine Formation

**FIGURE 1:**
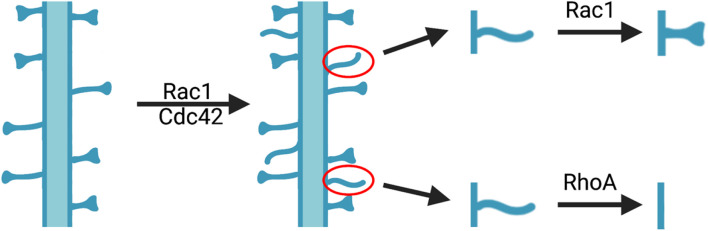
Role of Rho GTPases in modulating dendritic spine density and morphology. The activation of Rac1 and Cdc42 leads to increased immature spines, some of which will undergo morphological changes to obtain mature morphology through Rac1-dependent mechanisms, whereas others will be eliminated by RhoA-dependent processes.

Ample studies indicate that altering the activity of Rho GTPases can affect spinogenesis in developing neurons (Tashiro et al., 2000; Scott et al., 2003; Tashiro and Yuste, 2004; Zhang and Macara, 2006; Kang et al., 2008; Impey et al., 2010; Um et al., 2014; Orefice et al., 2016; Valdez et al., 2016; Moutin et al., 2017; Figure 1). Overaction of Rho GTPases in cultured primary neurons by cytotoxic necrotizing factor 1 (CNF-1) treatment resulted in enrichment in the actin cytoskeleton and dendritic processes (Diana et al., 2007). Suppression of Rac1 activity *in vitro* through the manipulations of dominant negative Rac1, the Rac1-GAP breakpoint cluster region protein (Bcr), or the Rac-GEF beta p21-activated protein kinase exchange factor (betaPIX), in cultured hippocampal neurons, decreased dendritic spine density (Nakayama et al., 2000; Penzes et al., 2003; Tashiro and Yuste, 2004; Zhang and Macara, 2006; Impey et al., 2010; Fiuza et al., 2013; Um et al., 2014). Genetic knockout (KO) of Rac1 in excitatory neurons in mice resulted in decreased spine and synapse numbers accompanied by increased postsynaptic density (PSD) (Haditsch et al., 2009). In contrast, elevating Rac1 activity *in vitro* through constitutively active Rac1 in cultured hippocampal slices leads to increased spine density but reduced spine size (Nakayama et al., 2000; Tashiro et al., 2000). In CA1 pyramidal neurons of Bcr KO mice, the spine density was higher compared to WT mice (Um et al., 2014). Similar to Rac1, conditional KO of Cdc42 in the hippocampus also resulted in decreased spine density and impaired spine size enlargement in response to glutamate uncaging (Kim et al., 2014). The expression of constitutively active Cdc42 increased spine density in cultured hippocampal neurons (Kang et al., 2008) and rescued the spine density deficit in LgDel mice, a model for 22q11.2 deletion syndrome with altered Cdc42 signaling (Moutin et al., 2017). The expression of loss-of-function Cdc42 mutants in *Drosophila* vertical system neurons led to similar deficits in spine density observed in mammals (Scott et al., 2003). In contrast to the effects of Rac1 and Cdc42, the expression of constitutively active RhoA in hippocampal neurons or slice cultures consistently resulted in simplified dendritic trees and reduced spine density (Nakayama et al., 2000; Tashiro et al., 2000; Impey et al., 2010). Inhibition of RhoA with C3 transferase, dominant negative RhoA, shRNA knockdown or other strategies, all resulted in increased spine density and impaired activity-dependent spine pruning (Tashiro et al., 2000; Kang et al., 2009; Impey et al., 2010; Orefice et al., 2016). It is important to note that the increased spines caused by RhoA inhibition or Rac1 activation appeared to be immature spines with filopodia-like or lamellipodia-like morphology (Nakayama et al., 2000; Tashiro et al., 2000). These results suggest that Rac1 and Cdc42 are involved in the induction and maintenance of spines, whereas RhoA plays a role in the elimination or pruning of immature spines. Therefore, the balance of Rac1/Cdc42 and RhoA activity may be critical for establishing and maintaining the homeostasis of dendritic spine density in the brain.

## Rho GTPases in Spine Morphology

Rho GTPases are critically involved in the regulation of spine morphology. For example, while spine density was found to decrease by blocking Rac1 activity, reduced spine head was also observed (Tashiro and Yuste, 2004), suggesting that Rac1 is essential for the enlargement or maturation of spines. The role of Rac1 in spine morphology was also shown in many other studies by increasing Rac1 activity via knockdown of upstream negative regulators (e.g., Bcr, alpha-chimaerin and p250GAP), expression of constitutively active Rac1, or neurotrophic factor BDNF treatment (Tashiro and Yuste, 2004; Wiens et al., 2005; Impey et al., 2010; Um et al., 2014; Orefice et al., 2016; Valdez et al., 2016). Interestingly, as mentioned earlier, Rac1 activation has also been shown to decrease spine size during early development, suggesting that the effect of Rac1 is age -dependent. In contrast to Rac1, the effect of RhoA on spine size is less clear (Tashiro and Yuste, 2004; Impey et al., 2010; Orefice et al., 2016).

Other than affecting basal spine morphology, Rho GTPases also participate in the activity-dependent remodeling of spine morphology. For example, during structural LTP (sLTP) induced by the glutamate uncaging at single spines of cultured hippocampal neurons, RhoA and Cdc42 activity was transiently increased and then decayed to a slight but stable elevation, coinciding with spine volume enlargement during sLTP (Murakoshi et al., 2011). Inhibition of RhoA activity via shRNA or C3 transferase, decreased both transient and sustained spine enlargement, while the inhibition of Cdc42 only impaired the sustained spine enlargement (Murakoshi et al., 2011). In this study, the investigators created novel sensors to measure RhoA and Cdc42 activity using the fluorescence resonance energy transfer (FRET) technique, allowing live imaging of the activity of these GTPases in live neurons. In a later study, similar sensors were made to image Rac1 activity during sLTP in hippocampal neurons, and it was found that Rac1 was also required for the transient and sustained spine enlargement (Hedrick et al., 2016). Interestingly, it was found that the active Rac1, Cdc42, and RhoA not only affected the morphological plasticity of activated spines, but also could diffuse to nearby spines and lower the threshold of sLTP induction in these spines, a phenomenon typically referred to as heterosynaptic plasticity or synaptic crosstalk. To explore the mechanism behind this crosstalk, the researchers examined the diffusivity of Rho GTPases during sLTP and showed that RhoA and Rac1 had a higher diffusion distance than Cdc42, that is, the active RhoA, and Rac1 diffused to the nearby spines whereas the active Cdc42 remained locally in the activated spine. Preventing the diffusion of active RhoA and Rac1 by an inhibitor protein that was fused with the microtubule binding domain of microtubule-associated protein 2 (MAP2) abolished the effect on heterosynaptic plasticity (Hedrick et al., 2016). Therefore, although Rac1, Cdc42, and RhoA all are involved in spine plasticity, they play differential roles, with spine-specific Cdc42 plus RhoA and Rac1 being all important for spine-specific homosynaptic sLTP, and the diffusion of the active RhoA and Rac1 being important for heterosynaptic sLTP.

## Rho GTPases in Synaptic Plasticity

**FIGURE 2:**
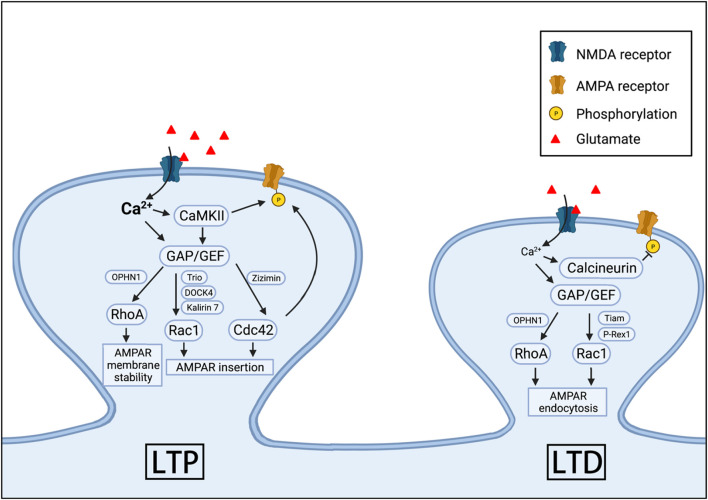
Rho GTPases in AMPAR expression, LTP and LTD. RhoA, Cdc42, and Rac1 can participate in the regulation of the synaptic insertion, internalization and membrane stability of AMPARs during LTP and LTD. These GPTases are activated by NMDARs or other surface proteins through various GAPs and GEFs.

In addition to affecting spine properties, many studies have shown that Rho GTPases are potent regulators of synaptic transmission and plasticity (Figure 2). Long-term potentiation (LTP) and long-term depression (LTD) are the two most extensively studied forms of long-lasting synaptic plasticity considered to be the basis for learning and memory (Bliss and Collingridge, 1993; Malenka and Bear, 2004; Citri and Malenka, 2008; Collingridge et al., 2010; Kandel et al., 2014). In the CA1 region of the hippocampus, the development of LTP and LTD depends on glutamatergic receptors in the postsynaptic density: α-amino-3-hydroxy-5-methyl-4-isoxazole-proponic acid receptors (AMPARs), and N-methyl-D-aspartate receptors (NMDARs) (Bliss and Collingridge, 1993; Kennedy, 2000; Bredt and Nicoll, 2003; Malenka and Bear, 2004; Huganir and Nicoll, 2013). Under resting membrane potential, AMPARs mediate the majority of synaptic transmission because NMDARs are inhibited by a voltage-dependent extracellular magnesium blockade. However, during intense neuronal activities and learning experience, activation of AMPARs causes sufficient postsynaptic depolarization that leads to the release of magnesium blockade of NMDARs and the influx of calcium through these receptors. Depending on the concentration and kinetics of calcium in the neurons, it can initiate two distinct signaling pathways via low-calcium-affinity kinase, CaMKII, or high-calcium-affinity phosphatase, calcineurin (Malenka and Bear, 2004; Collingridge et al., 2010; Huganir and Nicoll, 2013). These signaling molecules can then modulate many downstream effectors through various mechanisms to alter synaptic strength. For example, AMPAR can be directly phosphorylated by CaMKII to increase the channel conductance of AMPARs, thus potentiating synaptic transmission (Derkach et al., 1999; Kristensen et al., 2011; Huganir and Nicoll, 2013; Diering and Huganir, 2018). The regulation of AMPAR numbers at the postsynaptic membrane represents another key mechanism to modify synaptic strength during LTP and LTD. While the number of AMPARs at the synapse is maintained through constitutive recycling of the receptors for the stable basal synaptic transmission, the number can be drastically altered by activity-dependent endocytosis and exocytosis during LTD and LTP, respectively (Malinow and Malenka, 2002; Collingridge et al., 2004; Derkach et al., 2007; Anggono and Huganir, 2012). Rho GTPases may affect AMPARs at the postsynaptic membrane in several ways, including anchoring, clustering and trafficking of these receptors (Allison et al., 1998; Matsuzaki et al., 2001; Zhou et al., 2001; Wiens et al., 2005; Derkach et al., 2007; Zhou et al., 2011). For example, TC10, a member of Rho GTPases, was found to be involved in ADP-ribosylation factor 6 (Arf6)-mediated, clathrin-independent AMPAR constitutive translocation (Zheng et al., 2015). In this study, it was shown that TC10 existed in the Arf6-containing endosomes, and the expression of a dominant negative TC10 in the hippocampal neuron culture resulted in decreased surface/total AMPAR ratio, whereas constitutively active TC10 increased the ratio (Zheng et al., 2015). Cdc42 can also modulate AMPARs on the postsynaptic membrane. It was shown that Cdc42 participated in a signaling pathway that phosphorylates the AMPAR subunit, GluA1, at a novel phosphorylation site S863, which then facilitates the AMPAR expression on the postsynaptic surface (Hussain et al., 2015). In another study, it was found that LTP induction triggered cholesterol redistribution in the intracellular membrane, which was associated with the postsynaptic insertion of AMPARs and the activation of Cdc42 (Brachet et al., 2015). Cdc42 was required for the increased synaptic transmission induced by the cholesterol removal, and the expression of dominant negative Cdc42 abolished the increased AMPAR currents (Brachet et al., 2015). The effects of Rac1 on AMPAR trafficking appear to be complex. For example, in the hippocampal neurons of microtubule associated protein 1B (MAP1B) KO mice, endocytosis of AMPARs during LTD was impaired and this impairment was rescued by the overexpression of Rac1 (Benoist et al., 2013). Since MAP1B was known to facilitate the translocation of the Rac1-GEF protein, T-cell lymphoma invasion and metastasis protein 1 (Tiam1), to the synapses, the impaired endocytosis in MAP1B KO mice could be due to decreased Tiam1 expression at the synapse and hence reduced Rac1 activity. The role of Rac1 in AMPAR endocytosis during LTD is also supported by results from KO mice lacking the Rac1-GEF protein, phosphatidylinositol 3, 4, 5-trisphosphate-dependent Rac exchanger 1 (P-Rex1). It was reported that in hippocampal neurons of P-Rex1 KO mice, NMDA-induced reduction in surface AMPARs was impaired and overexpression of Rac1 rescued this impairment (Li et al., 2015). Furthermore, the pharmacological inhibition of Rac1 via NSC23766 in cultured hippocampal neurons increased AMPARs in the postsynaptic surface and decreased AMPARs in the cytoplasm (Glebov et al., 2015). In terms of LTP and AMPAR insertion, several studies have documented the essential role of Rac1 signaling through investigations of several Rac1-GEFs [e.g., Kalirin-7, dedicator of cytokinesis protein 4 (DOCK4), and triple functional domain protein (Trio)] where increased AMPAR expression at the synapse was shown with increased Rac1 activation (Xie et al., 2007; Sadybekov et al., 2017; Guo et al., 2021). Like Rac1 and Cdc42, RhoA also plays a role in the regulation of AMPAR trafficking. The activation of RhoA via knocking down its negative regulator was reported to be associated with decreased surface AMPARs (Shen et al., 2020), and impaired LTP was observed in the RhoA-GAP, oligophrenin-1 (OPHN1) KO mice (Kasri et al., 2009). The loss of OPHN1 also impaired activity-dependent AMPAR endocytosis, which was reversed by pharmacological inhibition of RhoA-ROCK signaling (Khelfaoui et al., 2009).

The results obtained from electrophysiological studies support the roles of Rho GTPases in synaptic transmission and plasticity. For example, increased Rac1 activity, either through KO/knockdown of Rac1 upstream GAP proteins such as alpha-chimaerin and BCR/ABR, or the expression of constitutively active Rac1 mutants, led to unstable theta-burst stimulation (TBS)-induced LTP that gradually decayed to the baseline response at the CA1 synapse (Oh et al., 2010; Liu et al., 2016, 2018; Lv et al., 2019). On the other hand, decreased Rac1 activity, either through the expression of dominant negative Rac1 mutants or the upstream GAP protein alpha-chimaerin, or through genetic deletion of MAP1B, resulted in more stable LTP (Glebov et al., 2015; Liu et al., 2016, 2018; Lv et al., 2019). One study, however, did observe impaired hippocampal LTP in Rac1 KO mice (Haditsch et al., 2009) although in this study, high-frequency stimulation (HFS) rather than TBS was used to induce LTP. LTD is also affected by manipulations of Rac1 activity. In MAP1B KO mice, low-frequency stimulation (LFS)-induced LTD was impaired (Benoist et al., 2013), suggesting that Rac1 is necessary for LTD. However, increased Rac1 activity by knocking out/down Rac1 GAP proteins (e.g., alpha-chimaerin and BCR/ABR) or overexpressing Rac1 had no effects on LFS-induced LTD (Oh et al., 2010; Benoist et al., 2013; Valdez et al., 2016), although in KO mice lacking kinesin family member 21B (Kif21B), a microtubule-dependent molecular motor that regulates the engulfment of the Rac1-GEF, ELMO1/DOCK complex and enhances Rac1 activity, was found to have unstable LFS-induced LTD without alterations in HFS-induced LTP at CA1 synapses (Morikawa et al., 2018). Although no clear role has emerged for Cdc42 in LTD, its involvement appeared to be critical for LTP, since HFS-induced LTP was absent in the hippocampus of Cdc42 KO mice (Kim et al., 2014). The role of RhoA in LTP was shown in hippocampal slices using RhoA shRNA or ROCK inhibitors, which affected the maintenance, but not induction, of TBS-induced LTP (Rex et al., 2009; Briz et al., 2015). Although the expression of RhoA dominant negative mutants had no effect on LFS-induced LTD (Benoist et al., 2013), overactivation of RhoA in OPHN1 KO mice impaired LTD (Khelfaoui et al., 2009), suggesting a role for RhoA in LTD.

In summary, despite that Rac1, Cdc42, and Rac1 are all involved in both insertion and internalization of AMPARs at the synapses, they appear to play distinct roles in LTP and LTD (Figure 2). While Cdc42 is required for LTP induction, RhoA is more important for LTP maintenance. Rac1 participates in destabilizing LTP and is also required for LTD expression. Clearly more studies are necessary to link specific roles of Rho GTPases in LTP and LTD with AMPAR trafficking and spine plasticity.

## Rho Signaling Pathways

As discussed earlier, the activity of Rho GTPases is tightly regulated by various GEF and GAP proteins (Govek et al., 2005). Many GEFs and GAPs have been identified and most of these proteins are expressed in the brain with unique spatial and temporal expression patterns (Moon and Zheng, 2003; Rossman et al., 2005). These GEFs and GAPs relay signals from various surface receptors, including NMDARs. Since each Rho GTPase can be modulated by multiple GEFs and GAPs, the spatial pattern of GAPs and GEFs determines the diversity and specificity of signals received by Rho GTPases. The importance of these GEFs and GAPs in the brain is evident from alterations in single GEF or GAP protein that can affect neuronal structure and function. For example, GEF-H1, a GEF for RhoA, negatively affects spine density and spine length of hippocampal neurons (Xie et al., 2007). Deletion of Kalirin-7 in mice resulted in schizophrenia-like phenotypes (Cahill et al., 2009). Changes in GAPs such as OPHN1 and SYNGAP1 were also found to result in cognitive deficits and autistic-like phenotypes that were accompanied by disrupted spine structure and function in both human and animal models (Newey et al., 2005; Kasri et al., 2009; Clement et al., 2012; Berryer et al., 2013; Ba and Nadif Kasri, 2017).

**FIGURE 3:**
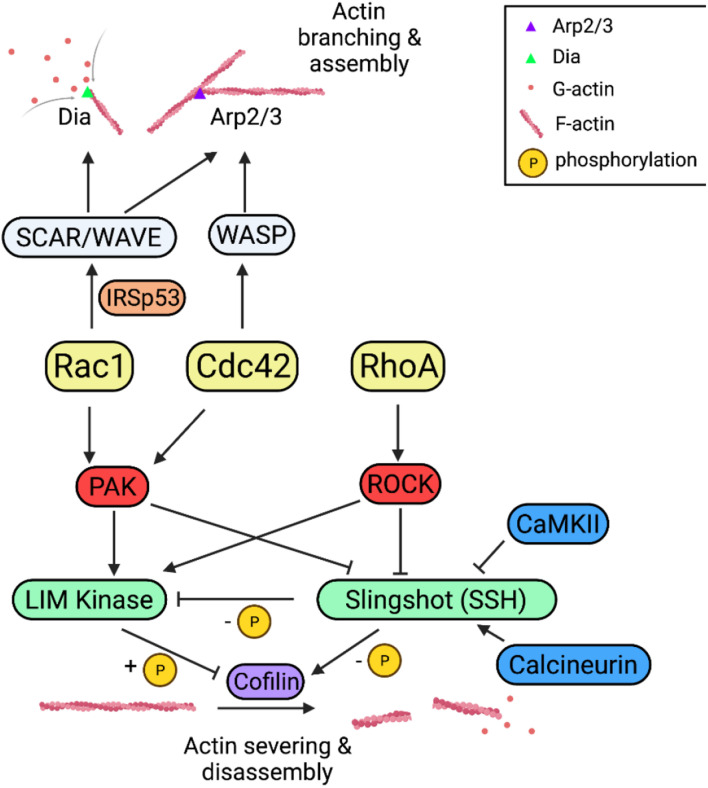
Rho GTPase signaling pathways in actin dynamics and synaptic function. Rac1, Cdc42, and RhoA regulate actin dynamics either through cofilin or Arp2/3 complex pathways. Rac1/Cdc42 and RhoA activate PAKs and ROCKs, respectively, which phosphorylate and activate cofilin kinase LIMKs and inhibit cofilin phosphatase SSH, both leading to inactivation of cofilin thus reduced actin disassembly. Rac1 and Cdc42 promote actin assembly through the Arp2/3 complex and the formin-related protein (Dia) via WAVE and WASP, respectively.

Downstream of Rho-GTPases are a chain of effector proteins that are involved in the regulation of the actin cytoskeleton (Figure 3). P21-activated kinase (PAKs) and the Rho-associated coiled-coil kinase (ROCK) are the two well-studied families of protein kinases activated by Rac1/Cdc42 and RhoA, respectively (Edwards et al., 1999; Maekawa et al., 1999). Both PAKs and ROCKs are potent regulators of spine morphogenesis and synaptic plasticity. For example, KO mice for PAK1/3 and ROCK2 displayed altered spine morphology, impaired LTP and LTD (Meng et al., 2005; Asrar et al., 2009; Zhou et al., 2009; Huang et al., 2011). PAK2 heterozygous mice exhibited reduced spine density and impaired LTP (Wang et al., 2018). Further downstream of ROCKs and PAKs are LIM-domain kinases (LIMKs), which directly phosphorylate and inhibit the activity of the actin depolymerization factor cofilin (Arber et al., 1998; Meng et al., 2002, 2003, 2004; Ben Zablah et al., 2020). Cofilin is an actin-binding protein that primarily functions to sever and depolymerize actin filaments (Bamburg, 1999; Andrianantoandro and Pollard, 2006; Bamburg and Bernstein, 2008; Ben Zablah et al., 2020). Similar to PAK1/3 and ROCK2 KO mice, LIMK1 KO mice displayed reduced spine size and impaired LTP (Meng et al., 2002, 2004). PAKs and ROCKs can also regulate the activity of cofilin through phosphorylating and inactivating the cofilin phosphatase, Slingshot homolog 1 (SSH1), which dephosphorylates and enhances cofilin function (Niwa et al., 2002; Soosairajah et al., 2005; Quassollo et al., 2015). SSH1 can directly interact with, dephosphorylate and inhibit LIMK1 (Soosairajah et al., 2005). Therefore, cofilin is an important converging point by which Rho GTPase regulate actin dynamics. Consistent with this, cofilin KO mice showed altered spine morphology and synaptic plasticity (Rust et al., 2010; Rust, 2015). In addition to affecting cofilin, both Rac1 and Cdc42 can control actin polymerization by regulating the Arp2/3 complex. The Arp2/3 complex is an actin nucleation factor to mediate the formation of branched actin network (Goley and Welch, 2006), which is thought to be essential for spine remodeling during spine growth (Hotulainen et al., 2009). The recruitment of the Arp2/3 complex to the actin filament is through the association with Wiskott-Aldrich syndrome protein (WASP) and WASP-family verprolin-homologous protein (WAVE) (Takenawa and Suetsugu, 2007). The neuronal (N)-WASP is activated by interaction with Cdc42, which relieves N-WASP from its intramolecular autoinhibition (Kim et al., 2000). Downregulation of N-WASP or deletion of its Arp2/3 binding region in hippocampal neurons resulted in decreased spine and synapse density (Wegner et al., 2008). Similar to WASP, loss of WAVE protein or manipulations of its phosphorylation status by cyclin-dependent kinase 5 (Cdk5) also caused deficits in spine formation and maintenance in mice (Soderling et al., 2003, 2007; Kim et al., 2006). WAVE is functionally similar to N-WASP, except that it lacks the binding domain for Rho-GTPases (Miki et al., 1998). Unlike Cdc42, Rac1 binds and activates WAVE through an adaptor protein, IRSp53, a substrate for the insulin receptor. The binding of WAVE protein to the SH3 domain of IRSp53 leads to the release of intramolecular inhibition of IRSp53 (Miki et al., 2000; Miki and Takenawa, 2002), and this results in the formation of a trimeric complex. Although Rac1/WAVE and Cdc42/N-WASP pathways converge on the Arp2/3 complex to modulate actin dynamics, they may play distinct roles in regulating memory. In a recent study on active forgetting of cold shock-sensitive or anesthesia-sensitive memory (ASM) and cold shock-insensitive or anesthesia-resistant memory (ARM) in *Drosophila*, it was found that the Arp2/3 complex was only required for Cdc42/WASP mediated ARM forgetting but not for Rac1/WAVE mediated ASM forgetting, and instead, Rac1/WAVE might function through another protein called Dia (Gao et al., 2019).

## Rho GTPases in Memory

Ample studies have indicated that Rho GTPases are involved in learning and memory. O’Kane et al. (2003) first reported that stimulation of the hippocampal CA1 region resulted in a noticeable increase in the activity of Rho GTPases. In a subsequent study using the protein toxin CNF-1 to increase the activity of Rho proteins, it was found that CNF-1 treated mice had an elevated level of actin filaments and enhanced freezing during a fear memory test and better performance in the Morris water maze test (Diana et al., 2007). However, this observed memory improvement may not be entirely attributable to the increased Rho GTPase activity because CNF-1 is not specific to the activation of the Rho proteins. For example, CNF-1 was reported to induce inflammatory responses and produce chemokines such as interleukin 8 (IL-8), monocyte chemoattractant protein-1 (MCP-1), and macrophage inflammatory protein 3 (MIP-3) (Munro et al., 2004). Similar to the effects on synaptic plasticity, different Rho GTPases play differential roles in memory. For example, RhoA signaling has been shown to be required for memory formation. Perturbations of the RhoA-ROCK signaling by intracerebral infusion of the ROCK inhibitor, Y27632, impaired conditioned place aversion (CPA) memory in rats, whereas the infusion of the Rac1 inhibitor, NSC23766, had no effect (Wang et al., 2013). Similar results were obtained in a conditioned place preference (CPP) memory test (Fakira et al., 2016) where it was found that RhoA signaling cascade was enhanced during morphine-induced CPP and that bilateral intracranial infusion of the ROCK inhibitor, H1152, completely prevented the formation of CPP. The suppression of RhoA activity through manipulations of p27 protein in cyclin-dependent kinase (CK) KO mice was also reported to be associated with deficits in hippocampus-dependent learning and memory (Kukalev et al., 2017). Although increased RhoA activity was shown to be crucial for synaptic plasticity and memory formation in adult animals, its role in early development seems to be different. It was reported that the exposure to the anesthetic drug, sevoflurane, in the early development could lead to persistent impairment of learning and memory in rodents (Lee et al., 2014), and that this effect was related to the activation of RhoA signaling pathway as the sevoflurane induced shortening of the dendritic protrusion length was blocked by the treatment of the ROCK inhibitor, Y27632 (Zimering et al., 2016). Bisphenol A (BPA), a chemical substance shown to impair the growth and development of the nervous system, was also reported to elevate the RhoA activity in hippocampal neurons and this was accompanied by decreased spike amplitudes and synaptic strength (Wang et al., 2020). Cdc42 is also involved in memory. For example, although neuron-specific Cdc42 conditional KO mice did not show alterations in short-term or long-term memory, they exhibited impairments in remote memory in both fear and spatial memory tests (Kim et al., 2014). In mouse nucleus accumbens (NAc), Cdc42 was activated by methamphetamine (METH) and viral expression of dominant negative and constitutively active Cdc42 impaired and enhanced METH-induced CPP memory, respectively (Tu et al., 2019). In *Drosophila* olfactory memory mediated by the mushroom bodies (MBs), ASM, and ARM were affected by different Rho GTPases (Shuai et al., 2010; Zhang et al., 2016; Gao et al., 2019). Expression of dominant negative Cdc42 in the MBs caused slower decay of ARM, whereas expression of constitutively active Cdc42 led to faster ARM decay (Zhang et al., 2016). The role of Rac1 in memory has been studied more extensively compared to Rho A and Cdc42. In Rac1 conditional KO mice where Rac1 was deleted in mature neurons, working memory in the delayed matching-to-place (DMP) water maze test was impaired (Haditsch et al., 2009), implicating Rac1 in memory acquisition but not in memory consolidation. However, subsequent studies using conditional expression of mutant Rac1 suggested that Rac1 is associated with memory forgetting instead of formation. This effect of Rac1 was initially demonstrated in *Drosophila* (Shuai et al., 2010) where controlled expression of dominant negative and constitutively active Rac1 mutants in the MBs using the Gal/UAS binary system delayed and facilitated aversive olfactory memory decay, respectively, without affecting memory acquisition (Shuai et al., 2010). Other than the passive memory decay, Rac1 was also investigated in the active memory forgetting using retroactive interference (RI) and reversal learning and shown to have similar effects as in the passive memory decay (Shuai et al., 2010). The effects of Rac1 in *Drosophila* memory forgetting are mediated by scribble scaffold protein (Cervantes-Sandoval et al., 2016). In mice, expression of constitutively active Rac1 hastened the memory forgetting in a novel object recognition (NOR) test whereas the expression of dominant negative Rac1 delayed forgetting (Liu et al., 2016). A similar role of Rac1 was shown in social memory, where increased Rac1 activity in the hippocampus by expressing constitutively active Rac1 accelerated the decay of social memory whereas inhibition of Rac1 activity delayed the social memory decay (Liu et al., 2018). In addition, it was found that Rac1 activity was elevated by social isolation and that socially isolated mice displayed faster social memory decay (Liu et al., 2018). Manipulations of Rac1 activity in the hippocampus using Rac1 inhibitor (NSC23766) and activator (CN04-A) were also reported to alter the maintenance of contextual fear memory in a similar fashion (Gan et al., 2016; Jiang et al., 2016). The role of Rac1 in forgetting is also supported by studies where Rac1 upstream regulators were manipulated. For example, KO mice lacking the Rac1 GAP protein, BCR/ABR, showed impaired spatial, and NOR memory by facilitating their decay (Oh et al., 2010). In KO mice deficient of another Rac1 GAP protein, ArhGAP15, the hyperactive Rac1 led to longer escaping latency during the reversal phases in the water maze test without affecting the learning process, as well as less freezing in both contextual and cued fear memory test (Zamboni et al., 2016). Recent studies have used optogenetic techniques that allow a more precise temporal and spatial control of Rac1 activity. For example, optical activation of photo-activable (PA) Rac1 in the amygdala was found to facilitate the cue-based long-term fear memory decay that was observed 24 h after training (Das et al., 2017). It was found that Rac1 activity increased after fear conditioning training and this increased Rac1 activity remained until fear memory disappeared, and that interfering Rac1 activity specifically during this period of time by optical stimulation of PA-Rac1 controlled the speed of memory decay (Lv et al., 2019).

In summary, while Cdc42 and RhoA seem to be involved the formation of memory, Rac1 is more important in memory forgetting. However, given that Rac1 KO in the excitatory neuron can also impair memory formation (Haditsch et al., 2009; Gao et al., 2015), the endogenous Rac1 level could be normally maintained at an intermediate level, which can then be up- or down- regulated to facilitate or slow down memory decay. Because the RI effect on memory and Rac1 activity was only evident 22 h (but not 8 h) after the initial training, there may exist a time window in which the Rac1 activity level is sensitive to endogenous modulation (Liu et al., 2016). This is consistent with the observations that activation of Rac1 during training but not after training impaired long-term memory, and that Rac1 inhibition during LTP induction, not after induction, impaired LTP (Martinez and Tejada-Simon, 2011; Das et al., 2017). Defining the molecular events during this critical window will be important to understand how memory is stored and consolidated.

## Rho GTPases in Brain Diseases

Considering the critical roles of Rho GTPases in spine formation and morphology, synaptic plasticity, and memory, it is not surprising that dysfunctions in these proteins are linked to various brain diseases, some of which will be briefly discussed below.

## Alzheimer’s Disease

Alzheimer’s disease (AD) is a neurodegenerative disease characterized by progressive loss of memory. The pathological hallmark of AD is the accumulation of extracellular amyloid plaques formed by amyloid beta (Ab) peptides and the aggregation of intracellular neurofibrillary tangles containing hyperphosphorylated Tau proteins (Hardy and Selkoe, 2002). Aβ peptides have been shown to induce abnormal assembly of actin bundles and the formation of cofilin-actin rods in neurons accompanied by neurite dystrophy and neuronal loss (Maloney and Bamburg, 2007), suggesting the involvement of Rho proteins. Evidence supporting the association between the elevated Rac1 activity and AD comes from both human and animal studies. Elevated Rac1 activity was observed in the hippocampus of post-mortem brains of AD patients as well as in APP/PS1 transgenic mice across different ages (3–9 months) and a fruit fly AD model (Wu et al., 2019). Suppression of Rac1 activity either by intragastric application of Rac1 inhibitor, Ehop-016, or by expression of dominant negative Rac1 rescued memory loss of APP/PS1 mice in water maze test, suggesting that increased Rac1 activity was responsible for the memory loss (Wu et al., 2019). Abnormalities in Rac1 were also reported in another AD mouse model in which elevated Rac1 activity was observed in the hippocampus of 6-week old 3xTg-AD mice, while the total Rac1 protein level was reduced in 7-month old 3xTg-AD mice (Borin et al., 2018). Contrary to the results obtained from the APP/PS1 mouse model, the intranasal treatment using constitutively active Rac1 rescued spine deficits in in 6.5-month-old 3xTg-AD mice (Borin et al., 2018). These results suggest that alterations in Rac1 may be age-dependent and affected by animal models. The effect of Rac1 in AD is likely mediated by changes in the actin cytoskeleton as Rac1 activation induced by Aβ peptides was associated with increased colocalization with actin filaments in hippocampal neurons, and the inhibition of Rac1 abolished the increased actin filaments (Mendoza-Naranjo et al., 2007). Increased Rac1 activity may also exacerbate AD symptoms through free radicals in astrocytes as Rac1 was required for Aβ-induced production of reactive oxygen species in these cells (Lee et al., 2002). The relationship between Aβ and Rac1 activity is not unidirectional; while Aβ treatment was shown to increase Rac1 activity, the expression of constitutively active Rac1 was able to increase the production of Aβ via increasing gamma-secretase-mediated cleavage of amyloid precursor protein (APP) (Gianni et al., 2003). Rac1 was also shown to enhance the transcription of APP via acting on the −233 to −41 bp region in the APP gene promoter, and inhibition of Rac1 activity by the Rac1 inhibitor, NSC23766, dominant negative Rac1, or siRNA knockdown, all reduced APP mRNA and protein level in the HEK293 cells (Wang et al., 2009).

Abnormal upregulation of Ccd42 was also shown in the brain of AD patients (Zhu et al., 2000). In addition, Aβ treatment of hippocampal neuronal culture induced the activation of Cdc42, along with Rac1, in a time- and dose-dependent manner (Mendoza-Naranjo et al., 2007). Similar to Rac1, the increased Cdc42 activity was colocalized with actin filaments and the inhibition of Cdc42 resulted in decreased actin filaments, supporting the involvement of actin changes in AD pathogenesis (Mendoza-Naranjo et al., 2007). In cultured hippocampal neurons from rats, Aβ-induced cofilin-actin rods were suppressed by dominant negative Cdc42 and enhanced by constitutively active Cdc42 (Davis et al., 2009). Although elevated Cdc42 level was found to be associated with AD, its downregulation in non-neuronal cells may also potentially contribute to AD. For example, KO mice of triggering receptor expressed on myeloid cells 2 (TREM2), a receptor predominantly expressed in the microglia and whose mutations were associated with increased risk of AD, showed alterations in Cdc42, and Rac1 signaling and their activator partially ameliorated impaired microglial migration in response to Aβ treatment (Rong et al., 2020).

In contrast to increased Rac1 and Cdc42 activity, decreased RhoA activity was found in the hippocampus of AD brains (Huesa et al., 2010). In addition, RhoA protein level was reduced in neuropil but increased in neurons that have neurofibrillary tangles. In the Tg2576 AD mouse model, altered distribution of RhoA was observed with a higher expression found in the dystrophic neurites but a lower level at the synapses (Huesa et al., 2010). In cultured PC12 cells, Aβ treatment caused activation of RhoA within 2 h of treatment and inhibition of RhoA by C3 transferase or dominant negative RhoA prevented Aβ-induced morphological alterations and neuronal death in cultured hippocampal neurons (Chacon et al., 2011). RhoA/ROCK signaling pathway is also involved in the production of Aβ peptides. In SH-SY5Y cells transfected with APP and in PDAPP AD mouse model, the amount of Aβ was decreased by the application of Y-27632 (Zhou et al., 2003). The role of RhoA in Aβ production could be mediated by Rac1 and Cdc42 through affecting the gamma-secretase cleavage of APP (Zhou et al., 2003). Unlike Rac1 and Cdc42 that can be palmitoylated on their C-terminus to facilitate their recruitment to the lipid raft on the plasma membrane where gamma-secretase was located, RhoA was found mainly in the non-raft fraction (Kumanogoh et al., 2001; Levental et al., 2010; Albanesi et al., 2020). Therefore, the effect of RhoA/ROCK pathway on APP processing and Aβ production is likely indirect. RhoA has also been shown to contribute to Aβ-mediated neurotoxicity by interfering with microtubule stability (Pianu et al., 2014). In non-neuronal cells such as cerebral endothelial cells and platelets, Aβ was found to activate RhoA/ROCK signaling and lead to the disruption of blood-brain barrier and activation of platelets, both of which are associated with AD (Sonkar et al., 2014; Park et al., 2017).

## Autism Spectrum Disorder

Autism spectrum disorder (ASD) is a neurodevelopmental disorder characterized by impaired social interaction and communication, and repetitive interests and behaviors. The pathogenesis of ASD is associated with genetic and environmental factors. To date, many genes have been identified that are linked to increased risk of developing ASD, and among these genes, 20 of them encode proteins involved in Rho signaling pathways (Guo et al., 2020), underscoring the importance of Rho proteins in the pathogenetic process of ASD. In addition, alterations in the activity of Rho GTPases are associated with many other genes linked to ASD. For example, duplication or deletion of the p11.2 region on chromosome 16 (16p11.2) is linked to ASD, and among the genes located in this region, potassium channel tetramerization domain containing 13 (kctd13) appeared to be important. kctd13 deficiency decreased synaptic transmission in the hippocampus and this was accompanied with increased RhoA activation. Inhibition of RhoA activity reversed the deficits caused by kctd13 deletion (Escamilla et al., 2017). Altered RhoA activity was also reported in the loss-of-function mutations of another key gene in 16p11.2, TAO kinase 2 (TAOK2) (Richter et al., 2019). TAOK2 deficiency caused dosage-dependent impairments in cognitive processes as well as abnormalities in neuronal structures and functions (Richter et al., 2019) and these changes were accompanied by reduced RhoA activity, and importantly pharmacological enhancement of RhoA activity restored abnormalities in neuronal structures (Richter et al., 2019). Alterations in Rac1 activity were also affected by several autism-risk gene mutations, including fragile X mental retardation 1 (Fmr1), neurexin 1 (Nrx1), neuroligin 4 (Nlg4), and tuberous sclerosis 1 (Tsc1). The mutations of these genes in *Drosophila* impaired reversal learning by affecting Rac1-mediated forgetting, and this impairment was rescued by overexpressing constitutively active Rac1 (Dong et al., 2016). Abnormal actin cytoskeleton was observed in the brain of ASD patients with compromised Rho signaling, suggesting that dysregulated actin may underlie the effects of Rho proteins in ASD (Griesi-Oliveira et al., 2018). In SH3 and multiple ankyrin repeat domains 3 (Shank3) KO mice, a widely used mouse model with autistic-like social deficit and repetitive behavior, reduced cortical actin filaments and Rac1/PAK activity were observed, which were rescued by reactivating Rac1 or inhibiting cofilin activity (Duffney et al., 2015).

### Fragile X Syndrome

Many Rho signaling proteins were reported to be linked to intellectual disability (Newey et al., 2005; van Galen and Ramakers, 2005). Fragile X syndrome (FXS) is the most commonly known single gene cause of ASD and intellectual disability. It is caused by a trinucleotide expansion within the Fmr1 gene on the X chromosome, resulting in an absence of FMR1 protein 1 (FMRP1) (Verkerk et al., 1991). Fmr1 KO mice displayed excessive immature spines, aberrant activation of Rac1, increased cofilin inactivation and actin polymerization (Pyronneau et al., 2017). Viral expression of constitutively active cofilin in the somatosensory cortex of Fmr1 KO mice or inhibition of PAK1, rescued cofilin changes and synaptic phenotypes in FXS mice (Pyronneau et al., 2017). Interestingly, although the overall baseline activity of Rac1 was increased in the brain of Fmr1 KO mice (Bongmba et al., 2011), the activation of Rac1 and PAK induced by TBS was impaired (Chen et al., 2010). Consistent with the impairment in activity-dependent Rac1 activation, inhibition of Rac1 rescued impaired LTP, elevated LTD and memory deficits in Fmr1 KO mice (Bongmba et al., 2011; Martinez and Tejada-Simon, 2018a,b).

## Summary

By using various approaches and techniques, including KO mouse models, pharmacological compounds and expression of mutant proteins, Rho GTPases and their effectors have been shown to play essential roles in the regulation of spine formation, spine morphology, receptor trafficking and synaptic plasticity, and learning and memory. It is important to note that the results obtained from these different approaches are not always consistent and this could be due to caveats associated with specific techniques used. For example, genetic KO mice may suffer from developmental compensation, whereas pharmacological reagents may have non-specific effects, both of which may complicate the interpretation of the data. Although members of Rho GTPases share many signaling molecules targeting the actin cytoskeleton, they often exert differential effects on synaptic structure and function. How these differential effects are achieved remains a challenging question to address. Downstream effector proteins targeting cellular processes other than actin dynamics may be important in this aspect. The temporal and spatial regulation of Rho GTPases also provides an important direction for future studies. In this regard, the use of photo-activatable Rac1 and cofilin may be particularly attractive (Wu et al., 2009; Das et al., 2017; Stone et al., 2019). Studies of Rho proteins in other brain cells, including astrocytes and microglia, are also of importance in understanding the role of Rho signaling in synaptic regulation. Given the extensive involvement of Rho signaling proteins in various brain diseases, understanding how these proteins are altered in the diseased brain continues to be a focus of future investigations. All these studies should aid the development of new strategies and molecular targets to treat related brain disorders.

## Author Contributions

HoZ, HiZ, and ZJ wrote and approved the manuscript. All authors contributed to the article and approved the submitted version.

## Conflict of Interest

The authors declare that the research was conducted in the absence of any commercial or financial relationships that could be construed as a potential conflict of interest.

## Publisher’s Note

All claims expressed in this article are solely those of the authors and do not necessarily represent those of their affiliated organizations, or those of the publisher, the editors and the reviewers. Any product that may be evaluated in this article, or claim that may be made by its manufacturer, is not guaranteed or endorsed by the publisher.

## References

[B1] Albanesi J. P. Barylko B. DeMartino G. N. Jameson D. M. (2020). Palmitoylated proteins in dendritic spine remodeling. *Front. Synap. Neurosci.* 12:22. 10.3389/fnsyn.2020.00022 32655390 PMC7325885

[B2] Allison D. W. Gelfand V. I. Spector I. Craig A. M. (1998). Role of actin in anchoring postsynaptic receptors in cultured hippocampal neurons: differential attachment of NMDA versus AMPA receptors. *J. Neurosci.* 18 2423–2436. 10.1523/jneurosci.18-07-02423.1998 9502803 PMC6793094

[B3] Alvarez V. A. Sabatini B. L. (2007). Anatomical and physiological plasticity of dendritic spines. *Ann. Rev. Neurosci.* 30 79–97. 10.1146/annurev.neuro.30.051606.094222 17280523

[B4] Andrianantoandro E. Pollard T. D. (2006). Mechanism of actin filament turnover by severing and nucleation at different concentrations of ADF/Cofilin. *Mol. Cell* 24 13–23. 10.1016/j.molcel.2006.08.006 17018289

[B5] Anggono V. Huganir R. L. (2012). Regulation of AMPA receptor trafficking and synaptic plasticity. *Curr. Opin. Neurobiol.* 22 461–469. 10.1016/j.conb.2011.12.006 22217700 PMC3392447

[B6] Arber S. Barbayannis F. A. Hanser H. Schnelder C. Stanyon C. A. Bernards O. (1998). Regulation of actin dynamics through phosphorylation of cofilin by LIM- kinase. *Nature* 393 805–809. 10.1038/31729 9655397

[B7] Asrar S. Meng Y. Zhou Z. Todorovski Z. Huang W. W. Jia Z. (2009). Regulation of hippocampal long-term potentiation by p21-activated protein kinase 1 (PAK1). *Neuropharmacology* 56 73–80. 10.1016/j.neuropharm.2008.06.055 18644395

[B8] Ba W. Nadif Kasri N. (2017). RhoGTPases at the synapse: an embarrassment of choice. *Small GTPases* 8 106–113. 10.1080/21541248.2016.1206352 27492682 PMC5464131

[B9] Bamburg J. R. (1999). Proteins of the ADF/cofilin family: essential regulators of actin dynamics. *Annu. Rev. Cell Dev. Biol.* 15 185–230. 10.1146/annurev.cellbio.15.1.185 10611961

[B10] Bamburg J. R. Bernstein B. W. (2008). ADF/Cofilin. *Curr. Biol.* 18 273–275. 10.1016/j.cub.2008.02.002 18397729

[B11] Ben Zablah Y. Merovitch N. Jia Z. (2020). FThe role of ADF/Cofilin in synaptic physiology and Alzheimer’s disease. *Front. Cell. Dev. Biol.* 8:594998. 10.3389/fcell.2020.594998 33282872 PMC7688896

[B12] Benoist M. Palenzuela R. Rozas C. Rojas P. Tortosa E. Morales B. (2013). MAP1B-dependent Rac activation is required for AMPA receptor endocytosis during long-term depression. *EMBO J.* 32 2287–2299. 10.1038/emboj.2013.166 23881099 PMC3746201

[B13] Berryer M. H. Hamdan F. F. Klitten L. L. Møller R. S. Carmant L. Schwartzentruber J. (2013). Mutations in SYNGAP1 cause intellectual disability, Autism, and a specific form of epilepsy by inducing haploinsufficiency. *Hum. Mutation* 34 385–394. 10.1002/humu.22248 23161826

[B14] Bliss T. V. P. Collingridge G. L. (1993). A synaptic model of memory: LTP in the hippocampus. *Nature* 361 31–39. 8421494 10.1038/361031a0

[B15] Bongmba O. Y. N. Martinez L. A. Elhardt M. E. Butler K. Tejada-Simon M. V. (2011). Modulation of dendritic spines and synaptic function by Rac1: a possible link to Fragile X syndrome pathology. *Brain Res.* 1399 79–95. 10.1016/j.brainres.2011.05.020 21645877 PMC3131096

[B16] Borin M. Saraceno C. Catania M. Lorenzetto E. Pontelli V. Paterlini A. (2018). Rac1 activation links tau hyperphosphorylation and Aß dysmetabolism in Alzheimer’s disease. *Acta Neuropathol. Commun.* 6:61. 10.1186/s40478-018-0567-4 30005699 PMC6045891

[B17] Bourne J. N. Harris K. M. (2008). Balancing structure and function at hippocampal dendritic spines. *Ann. Rev. Neurosci.* 31 47–67. 10.1146/annurev.neuro.31.060407.125646 18284372 PMC2561948

[B18] Bourne J. Harris K. M. (2007). Do thin spines learn to be mushroom spines that remember? *Curr. Opin. Neurobiol.* 17 381–386. 10.1016/j.conb.2007.04.009 17498943

[B19] Brachet A. Norwood S. Brouwers J. F. Palomer E. Helms J. B. Dotti C. G. (2015). LTP-triggered cholesterol redistribution activates Cdc42 and drives AMPA receptor synaptic delivery. *J. Cell Biol.* 208 791–806. 10.1083/jcb.201407122 25753037 PMC4362467

[B20] Bredt D. S. Nicoll R. A. (2003). AMPA receptor trafficking at excitatory synapses. *Neuron* 40 361–379. 10.1016/S0896-6273(03)00640-8 14556714

[B21] Briz V. Zhu G. Wang Y. Liu Y. Avetisyan M. Bi X. (2015). Activity-dependent rapid local RhoA synthesis is required for hippocampal synaptic plasticity. *J. Neurosci.* 35 2269–2282. 10.1523/JNEUROSCI.2302-14.2015 25653381 PMC4315844

[B22] Cahill M. E. Xie Z. Day M. Photowala H. Barbolina M. V. Miller C. A. (2009). Kalirin regulates cortical spine morphogenesis and disease-related behavioral phenotypes. *Proc. Natl. Acad. Sci. U.S.A.* 106:16890. 10.1073/pnas.0909813106 PMC2722269 19625617

[B23] Cervantes-Sandoval I. Chakraborty M. MacMullen C. Davis R. L. (2016). Scribble scaffolds a Signalosome for active forgetting. *Neuron* 90 1230–1242. 10.1016/j.neuron.2016.05.010 27263975 PMC4926877

[B24] Chacon P. J. Garcia-Mejias R. Rodriguez-Tebar A. (2011). Inhibition of RhoA GTPase and the subsequent activation of PTP1B protects cultured hippocampal neurons against amyloid β toxicity. *Mol. Neurodegen.* 6:14. 10.1186/1750-1326-6-14 21294893 PMC3038970

[B25] Chen L. Y. Rex C. S. Babayan A. H. Kramár E. A. Lynch G. Gall C. M. (2010). Physiological activation ofsynaptic Rac>PAK (p-21 activated kinase) signaling is defective in a mouse model offragile X syndrome. *J. Neurosci.* 30 10977–10984. 10.1523/JNEUROSCI.1077-10.2010 20720104 PMC2929244

[B26] Chidambaram S. B. Rathipriya A. G. Bolla S. R. Bhat A. Ray B. Mahalakshmi A. M. (2019). Dendritic spines: revisiting the physiological role. *Prog. Neuro Psychopharmacol. Biol. Psychiatry* 92 161–193. 10.1016/j.pnpbp.2019.01.005 30654089

[B27] Cingolani L. A. Goda Y. (2008). Actin in action: the interplay between the actin cytoskeleton and synaptic efficacy. *Nat. Rev. Neurosci.* 9 344–356. 10.1038/nrn2373 18425089

[B28] Citri A. Malenka R. C. (2008). Synaptic plasticity: multiple forms, functions, and mechanisms. *Neuropsychopharmacology* 33 18–41. 10.1038/sj.npp.1301559 17728696

[B29] Clement J. P. Aceti M. Creson T. K. Ozkan E. D. Shi Y. Reish N. J. (2012). Pathogenic SYNGAP1 mutations impair cognitive development by disrupting maturation of dendritic spine synapses. *Cell* 151 709–723. 10.1016/j.cell.2012.08.045 23141534 PMC3500766

[B30] Collingridge G. L. Isaac J. T. R. Yu T. W. (2004). Receptor trafficking and synaptic plasticity. *Nat. Rev. Neurosci.* 5 952–962. 10.1038/nrn1556 15550950

[B31] Collingridge G. L. Peineau S. Howland J. G. Wang Y. T. (2010). Long-term depression in the CNS. *Nat. Rev. Neurosci.* 11 459–473. 10.1038/nrn2867 20559335

[B32] Das A. Dines M. Alapin J. M. Lamprecht R. (2017). Affecting long-term fear memory formation through optical control of Rac1 GTPase and PAK activity in lateral amygdala. *Sci. Rep.* 7:13930. 10.1038/s41598-017-13674-9 29066727 PMC5655381

[B33] Davis R. C. Maloney M. T. Minamide L. S. Flynn K. C. Stonebraker M. A. Bamburg J. R. (2009). Mapping cofilin-actin rods in stressed hippocampal slices and the role of cdc42 in amyloid-beta-induced rods. *J. Alzheimers Dis.* 18 35–50. 10.3233/JAD-2009-1122 19542631 PMC4477627

[B34] Derkach V. A. Oh M. C. Guire E. S. Soderling T. R. (2007). Regulatory mechanisms of AMPA receptors in synaptic plasticity. *Nat. Rev. Neurosci.* 8 101–113. 10.1038/nrn2055 17237803

[B35] Derkach V. Barria A. Soderling T. R. (1999). Ca2+/calmodulin-kinase II enhances channel conductance of α-amino-3-hydroxy-5-methyl-4-isoxazolepropionate type glutamate receptors. *Proc. Natl. Acad. Sci. U.S.A.* 96 3269–3274. 10.1073/pnas.96.6.3269 10077673 PMC15931

[B36] Diana G. Valentini G. Travaglione S. Falzano L. Pieri M. Zona C. (2007). Enhancement of learning and memory after activation of cerebral Rho GTPases. *Proc. Natl. Acad. Sci. U.S.A.* 104 636–641. 10.1073/pnas.0610059104 17202256 PMC1761909

[B37] Diering G. H. Huganir R. L. (2018). The AMPA receptor code of synaptic plasticity. *Neuron* 100 314–329. 10.1016/j.neuron.2018.10.018 30359599 PMC6214363

[B38] Dong T. He J. Wang S. Wang L. Cheng Y. Zhong Y. (2016). Inability to activate Rac1-dependent forgetting contributes to behavioral inflexibility in mutants of multiple autism-risk genes. *Proc. Natl. Acad. Sci. U.S.A.* 113 7644–7649. 10.1073/pnas.1602152113 27335463 PMC4941477

[B39] Duffney L. J. Zhong P. Wei J. Matas E. Cheng J. Qin L. (2015). Autism-like deficits in Shank3-deficient mice are rescued by targeting actin regulators. *Cell Rep.* 11 1400–1413. 10.1016/j.celrep.2015.04.064 26027926 PMC4464902

[B40] Edwards D. C. Sanders L. C. Bokoch G. M. Gill G. N. (1999). Activation of LIM-kinase by Pak1 couples Rac/Cdc42 GTPase signalling to actin cytoskeletal dynamics. *Nat. Cell Biol.* 1 253–259. 10559936 10.1038/12963

[B41] Escamilla C. O. Filonova I. Walker A. K. Xuan Z. X. Holehonnur R. Espinosa F. (2017). Kctd13 deletion reduces synaptic transmission via increased RhoA. *Nature* 551 227–231. 10.1038/nature24470 29088697 PMC5787033

[B42] Etienne-Manneville S. Hall A. (2002). Rho GTPases in cell biology. *Nature* 420 629–635. 10.1038/nature01148 12478284

[B43] Fakira A. K. Massaly N. Cohensedgh O. Berman A. Morón J. A. (2016). Morphine-associated contextual cues induce structural plasticity in Hippocampal CA1 pyramidal neurons. *Neuropsychopharmacology* 41 2668–2678. 10.1038/npp.2016.69 27170097 PMC5026734

[B44] Fiala J. C. Feinberg M. Popov V. Harris K. M. (1998). Synaptogenesis via dendritic filopodia in developing hippocampal area CA1. *J. Neurosci.* 18 8900–8911. 10.1523/JNEUROSCI.18-21-08900.1998 9786995 PMC6793554

[B45] Fiuza M. González-González I. Pérez-Otaño I. (2013). GluN3A expression restricts spine maturation via inhibition of GIT1/Rac1 signaling. *Proc. Natl. Acad. Sci. U.S.A.* 110 20807–20812. 10.1073/pnas.1312211110 24297929 PMC3870762

[B46] Gan P. Ding Z. Y. Gan C. Mao R. R. Zhou H. Xu L. (2016). Corticosterone regulates fear memory via Rac1 activity in the hippocampus. *Psychoneuroendocrinology* 71 86–93. 10.1016/j.psyneuen.2016.05.011 27249795

[B47] Gao Q. Yao W. Wang J. Yang T. Liu C. Tao Y. (2015). Post-training activation of Rac1 in the basolateral amygdala is required for the formation of both short-term and long-term auditory fear memory. *Front. Mol. Neurosci.* 8:65. 10.3389/fnmol.2015.00065 26582975 PMC4631819

[B48] Gao Y. Shuai Y. Zhang X. Peng Y. Wang L. He J. (2019). Genetic dissection of active forgetting in labile and consolidated memories in *Drosophila*. *Proc. Natl. Acad. Sci. U.S.A.* 116 21191–21197. 10.1073/pnas.1903763116 31488722 PMC6800343

[B49] Gianni D. Zambrano N. Bimonte M. Minopoli G. Mercken L. Talamo F. (2003). Platelet-derived growth factor induces the β-γ-secretase-mediated cleavage of Alzheimer’s amyloid precursor protein through a Src-Rac-dependent pathway. *J. Biol. Chem.* 278 9290–9297. 10.1074/jbc.M211899200 12645527

[B50] Glebov O. O. Tigaret C. M. Mellor J. R. Henley J. M. (2015). Clathrin-independent trafficking of AMPA receptors. *J. Neurosci.* 35 4830–4836. 10.1523/JNEUROSCI.3571-14.2015 25810514 PMC4389590

[B51] Goley E. D. Welch M. D. (2006). The ARP2/3 complex: an actin nucleator comes of age. *Nat. Rev. Mol. Cell Biol.* 7 713–726. 10.1038/nrm2026 16990851

[B52] Govek E. E. Newey S. E. Van Aelst L. (2005). The role of the Rho GTPases in neuronal development. *Genes Dev.* 19 1–49. 10.1101/gad.1256405 15630019

[B53] Gray E. G. (1959). Axo-somatic and axo-dendritic synapses of the cerebral cortex: an electron microscope study. *J. Anat.* 93 420–433. 10.1016/B978-0-12-801426-4.05001-X 13829103 PMC1244535

[B54] Griesi-Oliveira K. Suzuki A. M. Alves A. Y. Mafra A. C. C. N. Yamamoto G. L. Ezquina S. (2018). Actin cytoskeleton dynamics in stem cells from autistic individuals. *Sci. Rep.* 8:11138. 10.1038/s41598-018-29309-6 30042445 PMC6057935

[B55] Guo D. Peng Y. Wang L. Sun X. Wang X. Liang C. (2021). Autism-like social deficit generated by Dock4 deficiency is rescued by restoration of Rac1 activity and NMDA receptor function. *Mol. Psychiatry* 26 1505–1519. 10.1038/s41380-019-0472-7 31388105 PMC8159750

[B56] Guo D. Yang X. Shi L. (2020). Rho GTPase regulators and effectors in autism spectrum disorders: animal models and insights for therapeutics. *Cells* 9:835. 10.3390/cells9040835 32244264 PMC7226772

[B57] Haditsch U. Leone D. P. Farinelli M. Chrostek-Grashoff A. Brakebusch C. Mansuy I. M. (2009). A central role for the small GTPase Rac1 in hippocampal plasticity and spatial learning and memory. *Mol. Cell. Neurosci.* 41 409–419. 10.1016/j.mcn.2009.04.005 19394428 PMC2705331

[B58] Hardy J. Selkoe D. J. (2002). The amyloid hypothesis of Alzheimer’s disease: progress and problems on the road to therapeutics. *Science* 297 353–356. 10.1126/science.1072994 12130773

[B59] Harris K. M. Jensen F. E. Tsao B. (1992). Three-dimensional structure of dendritic spines and synapses in rat hippocampus (CA1) at postnatal day 15 and adult ages: implications for the maturation of synaptic physiology and long-term potentiation. *J. Neurosci.* 12 2685–2705. 1613552 10.1523/JNEUROSCI.12-07-02685.1992 PMC6575840

[B60] Hass K. T. Compans B. Letellier M. Bartol T. Grillo-Bosch D. Sejnowski T. (2018). Pre-post synaptic alignment through neuroligin tunes synaptic transmission efficiency. *ELife* 7:189407. 10.7554/eLife.31755 30044218 PMC6070337

[B61] Hedrick N. G. Harward S. C. Hall C. E. Murakoshi H. McNamara J. O. Yasuda R. (2016). Rho GTPase complementation underlies BDNF-dependent homo- and heterosynaptic plasticity. *Nature* 538 104–108. 10.1038/nature19784 27680697 PMC5361895

[B62] Holtmaat A. Svoboda K. (2009). Experience-dependent structural synaptic plasticity in the mammalian brain. *Nat. Rev. Neurosci.* 10 647–658. 10.1038/nrn2699 19693029

[B63] Hotulainen P. Hoogenraad C. C. (2010). Actin in dendritic spines: connecting dynamics to function. *J. Cell Biol.* 189 619–629. 10.1083/jcb.201003008 20457765 PMC2872912

[B64] Hotulainen P. Llano O. Smirnov S. Tanhuanpää K. Faix J. Rivera C. (2009). Defning mechanisms of actin polymerization and depolymerization during Dendritic spine morphogenesis. *J. Cell Biol.* 185 323–339. 10.1083/jcb.200809046 19380880 PMC2700375

[B65] Huang W. Zhou Z. Asrar S. Henkelman M. Xie W. Jia Z. (2011). p21-Activated kinases 1 and 3 control brain size through coordinating neuronal complexity and synaptic properties. *Mol. Cell. Biol.* 31 388–403. 10.1128/mcb.00969-10 21115725 PMC3028630

[B66] Huesa G. Baltrons M. A. Gómez-Ramos P. Morán A. García A. Hidalgo J. (2010). Altered distribution of RhoA in Alzheimer’s disease and AβPP overexpressing mice. *J. Alzheimers Dis.* 19 37–56. 10.3233/JAD-2010-1203 20061625

[B67] Huganir R. L. Nicoll R. A. (2013). AMPARs and synaptic plasticity: the last 25 years. *Neuron* 80 704–717. 10.1016/j.neuron.2013.10.025 24183021 PMC4195488

[B68] Hussain N. K. Thomas G. M. Luo J. Huganir R. L. (2015). Regulation of AMPA receptor subunit GluA1 surface expression by PAK3 phosphorylation. *Proc. Natl. Acad. Sci. U.S.A.* 112 E5883–E5890. 10.1073/pnas.1518382112 26460013 PMC4629353

[B69] Impey S. Davare M. Lasiek A. Fortin D. Ando H. Varlamova O. (2010). An activity-induced microRNA controls dendritic spine formation by regulating Rac1-PAK signaling. *Mol. Cell. Neurosci.* 43 146–156. 10.1016/j.mcn.2009.10.005 19850129 PMC2818337

[B70] Jiang L. Mao R. Zhou Q. Yang Y. Cao J. Ding Y. (2016). Inhibition of Rac1 activity in the hippocampus impairs the forgetting of contextual fear memory. *Mol. Neurobiol.* 53 1247–1253. 10.1007/s12035-015-9093-6 25613020

[B71] Kandel E. R. Dudai Y. Mayford M. R. (2014). The molecular and systems biology of memory. *Cell* 157 163–186. 10.1016/j.cell.2014.03.001 24679534

[B72] Kang M. G. Guo Y. Huganir R. L. (2009). AMPA receptor and GEF-H1/Lfc complex regulates dendritic spine development through RhoA signaling cascade. *Proc. Natl. Acad. Sci. U.S.A.* 106 3549–3554. 10.1073/pnas.0812861106 19208802 PMC2638734

[B73] Kang R. Wan J. Arstikaitis P. Takahashi H. Huang K. Bailey A. O. (2008). Neural palmitoyl-proteomics reveals dynamic synaptic palmitoylation. *Nature* 456 904–909. 10.1038/nature07605 19092927 PMC2610860

[B74] Kasai H. Fukuda M. Watanabe S. Hayashi-Takagi A. Noguchi J. (2010). Structural dynamics of dendritic spines in memory and cognition. *Trends Neurosci.* 33 121–129. 10.1016/j.tins.2010.01.001 20138375

[B75] Kasri N. N. Nakano-Kobayashi A. Malinow R. Li B. Van Aelst L. (2009). The Rho-linked mental retardation protein oligophrenin-1 controls synapse maturation and plasticity by stabilizing AMPA receptors. *Genes Dev.* 23 1289–1302. 10.1101/gad.1783809 19487570 PMC2701582

[B76] Kennedy M. B. (2000). Signal-processing machines at the postsynaptic density. *Science* 290 750–754. 10.1126/science.290.5492.750 11052931

[B77] Khelfaoui M. Pavlowsky A. Powell A. D. Valnegri P. Cheong K. W. Blandin Y. (2009). Inhibition of RhoA pathway rescues the endocytosis defects in Oligophrenin1 mouse model of mental retardation. *Hum. Mol. Genet.* 18 2575–2583. 10.1093/hmg/ddp189 19401298 PMC2701329

[B78] Kim A. S. Kakalis L. T. Abdul-Manan N. Liu G. A. Rosen M. K. (2000). Autoinhibition and activation mechanisms of the wiskott-aldrich syndrome protein. *Nature* 404 151–158. 10.1038/35004513 10724160

[B79] Kim I. H. Wang H. Soderling S. H. Yasuda R. (2014). Loss of Cdc42 leads to defects in synaptic plasticity and remote memory recall. *ELife* 3:e02839. 10.7554/eLife.02839 25006034 PMC4115656

[B80] Kim Y. Sung J. Y. Ceglia I. Lee K. W. Ahn J. H. Halford J. M. (2006). Phosphorylation of WAVE1 regulates actin polymerization and dendritic spine morphology. *Nature* 442 814–817. 10.1038/nature04976 16862120

[B81] Korobova F. Svitkina T. (2010). Molecular architecture of synaptic actin cytoskeleton in hippocampal neurons reveals a mechanism of dendritic spine morphogenesis. *Mol. Biol. Cell* 21 1033–1046. 10.1091/mbc.E09 19889835 PMC2801710

[B82] Kristensen A. S. Jenkins M. A. Banke T. G. Schousboe A. Makino Y. Johnson R. C. (2011). Mechanism of Ca2+/calmodulin-dependent kinase II regulation of AMPA receptor gating. *Nat. Neurosci.* 14 727–735. 10.1038/nn.2804 21516102 PMC3102786

[B83] Kukalev A. Ng Y. M. Ju L. Saidi A. Lane S. Mondragon A. (2017). Deficiency of cks1 leads to learning and long-term memory defects and p27 dependent formation of neuronal cofilin aggregates. *Cereb. Cortex* 27 11–23. 10.1093/cercor/bhw354 28365778 PMC5939225

[B84] Kumanogoh H. Miyata S. Sokawa Y. Maekawa S. (2001). Biochemical and morphological analysis on the localization of Rac1 in neurons. *Neurosci. Res.* 39 189–196. 10.1016/S0168-0102(00)00211-X 11223464

[B85] Lee B. H. Chan J. T. Hazarika O. Vutskits L. Sall J. W. (2014). Early exposure to volatile anesthetics impairs long-term associative learning and recognition memory. *PLoS One* 9:e105340. 10.1371/journal.pone.0105340 25165850 PMC4148240

[B86] Lee M. You H. Cho S. Woo C. Yoo M. Joe E. (2002). Implication of the small GTPase Rac1 in the generation of reactive oxygen species in response to beta-amyloid in C6 astroglioma cells. *Biochem. J.* 366 937–943. 10.1042/BJ20020453 12038964 PMC1222817

[B87] Levental I. Lingwood D. Grzybek M. Coskun Ü Simons K. (2010). Palmitoylation regulates raft affinity for the majority of integral raft proteins. *Proc. Natl. Acad. Sci. U.S.A.* 107 22050–22054. 10.1073/pnas.1016184107 21131568 PMC3009825

[B88] Li J. Chai A. Wang L. Ma Y. Wu Z. Yu H. (2015). Synaptic P-Rex1 signaling regulates hippocampal longterm depression and autism-like social behavior. *Proc. Natl. Acad. Sci. U.S.A.* 112 E6964–E6972. 10.1073/pnas.1512913112 26621702 PMC4687539

[B89] Liu Y. Du S. Lv L. Lei B. Shi W. Tang Y. (2016). Hippocampal activation of Rac1 regulates the forgetting of object recognition memory. *Curr. Biol.* 26 2351–2357. 10.1016/j.cub.2016.06.056 27593377

[B90] Liu Y. Lv L. Wang L. Zhong Y. (2018). Social isolation induces Rac1-dependent forgetting of social memory. *Cell Rep.* 25 288–295.e3. 10.1016/j.celrep.2018.09.033 30304669

[B91] Lv L. Liu Y. Xie J. Wu Y. Zhao J. Li Q. (2019). Interplay between α2-chimaerin and Rac1 activity determines dynamic maintenance of long-term memory. *Nat. Commun.* 10:5313. 10.1038/s41467-019-13236-9 31757963 PMC6876637

[B92] Maekawa M. Ishizaki T. Boku S. Watanabe N. Fujita A. Iwamatsu A. (1999). Signaling from Rho to the actin cytoskeleton through protein kinases ROCK and LIM-kinase. *Science* 285 895–898. 10.1126/science.285.5429.895 10436159

[B93] Malenka R. C. Bear M. F. (2004). LTP and LTD: an embarrassment of riches. *Neuron* 44 5–21. 10.1016/j.neuron.2004.09.012 15450156

[B94] Malinow R. Malenka R. C. (2002). AMPA receptor trafficking and synaptic plasticity. *Ann. Rev. Neurosci.* 25 103–126. 10.1146/annurev.neuro.25.112701.142758 12052905

[B95] Maloney M. T. Bamburg J. R. (2007). Cofilin-mediated neurodegeneration in Alzheimer’s disease and other amyloidopathies. *Mol. Neurobiol.* 35 21–43. 10.1007/BF02700622 17519504

[B96] Martinez L. A. Tejada-Simon M. V. (2011). Pharmacological inactivation of the small GTPase Rac1 impairs long-term plasticity in the mouse hippocampus. *Neuropharmacology* 61 305–312. 10.1016/j.neuropharm.2011.04.017 21569781 PMC3106418

[B97] Martinez L. A. Tejada-Simon M. V. (2018a). Increased training intensity induces proper membrane localization of actin remodeling proteins in the hippocampus preventing cognitive deficits: implications for fragile X syndrome. *Mol. Neurobiol.* 55 4529–4542. 10.1007/s12035-017-0666-4 28688003

[B98] Martinez L. A. Tejada-Simon M. V. (2018b). Pharmacological rescue of hippocampal fear learning deficits in fragile X syndrome. *Mol. Neurobiol.* 55 5951–5961. 10.1007/s12035-017-0819-5 29128904

[B99] Matsuzaki M. Ellis-Davies G. C. R. Nemoto T. Miyashita Y. Iino M. Kasai H. (2001). Dendritic spine geometry is critical for AMPA receptor expression in hippocampal CA1 pyramidal neurons. *Nat. Neurosci.* 4 1086–1092. 10.1038/nn736 11687814 PMC4229049

[B100] Mendoza-Naranjo A. Gonzalez-Billault C. Maccioni R. B. (2007). Aβ1-42 stimulates actin polymerization in hippocampal neurons through Rac1 and Cdc42 Rho GTPases. *J. Cell Sci.e* 120 279–288. 10.1242/jcs.03323 17200137

[B101] Meng J. Meng Y. Hanna A. Janus C. Jia Z. (2005). Abnormal long-lasting synaptic plasticity and cognition in mice lacking the mental retardation gene Pak3. *J. Neurosci.* 25 6641–6650. 10.1523/JNEUROSCI.0028-05.2005 16014725 PMC6725420

[B102] Meng Y. Takahashi H. Meng J. Zhang Y. Lu G. Asrar S. (2004). Regulation of ADF/cofilin phosphorylation and synaptic function by LIM-kinase. *Neuropharmacology* 47 746–754. 10.1016/j.neuropharm.2004.06.030 15458846

[B103] Meng Y. Zhang Y. Tregoubov V. Falls D. L. Jia Z. (2003). Regulation of spine morphology and synaptic function by LIMK and the actin cytoskeleton. *Rev. Neurosci.* 14 233–240. 10.1515/REVNEURO.2003.14.3.233 14513866

[B104] Meng Y. Zhang Y. Tregoubov V. Janus C. Cruz L. Jackson M. (2002). Abnormal spine morphology and enhanced LTP in LIMK-1 knockout mice. *Neuron* 35 121–133. 10.1016/S0896-6273(02)00758-4 12123613

[B105] Miki H. Takenawa T. (2002). WAVE2 serves a functional partner of IRSp53 by regulating its interaction with Rac. *Biochem. Biophys. Res. Commun.* 293 93–99. 10.1016/S0006-291X(02)00218-8 12054568

[B106] Miki H. Suetsugu S. Takenawa T. (1998). WAVE, a novel WASP-family protein involved in actin reorganization induced by Rac. *EMBO J.* 17 6932–6941. 10.1093/emboj/17.23.6932 9843499 PMC1171041

[B107] Miki H. Yamaguchi H. Suetsugu S. Takenawa T. (2000). IRSp53 is an essential intermediate between Rac and WAVE in the regulation of membrane ruffling. *Nature* 408 732–735. 10.1038/35047107 11130076

[B108] Missler M. Südhof T. C. Biederer T. (2012). Synaptic cell adhesion. *Cold Spring Harb. Perspect. Biol.* 4:a005694. 10.1101/cshperspect.a005694 22278667 PMC3312681

[B109] Moon S. Y. Zheng Y. (2003). Rho GTPase-activating proteins in cell regulation. *Trends Cell Biol.* 13 13–22. 10.1016/S0962-8924(02)00004-1 12480336

[B110] Morikawa M. Tanaka Y. Cho H. S. Yoshihara M. Hirokawa N. (2018). The molecular motor KIF21B mediates synaptic plasticity and fear extinction by terminating Rac1 activation. *Cell Rep.* 23 3864–3877. 10.1016/j.celrep.2018.05.089 29949770

[B111] Moutin E. Nikonenko I. Stefanelli T. Wirth A. Ponimaskin E. De Roo M. (2017). Palmitoylation of cdc42 promotes spine stabilization and rescues spine density deficit in a mouse model of 22q11.2 deletion syndrome. *Cereb. Cortex* 27 3618–3629. 10.1093/cercor/bhw183 27365300

[B112] Munro P. Flatau G. Doye A. Boyer L. Oregioni O. Mege J. L. (2004). Activation and proteasomal degradation of Rho GTPases by cytotoxic necrotizing factor-1 elicit a controlled inflammatory response. *J. Biol. Chem.* 279 35849–35857. 10.1074/jbc.M401580200 15152002

[B113] Murakoshi H. Wang H. Yasuda R. (2011). Local, persistent activation of Rho GTPases during plasticity of single dendritic spines. *Nature* 472 100–106. 10.1038/nature09823 21423166 PMC3105377

[B114] Nakayama A. Y. Harms M. B. Luo L. (2000). Small GTPases Rac and Rho in the maintenance of dendritic spines and branches in hippocampal pyramidal neurons. *J. Neurosci.* 20 5329–5338. 10.1523/jneurosci.20-14-05329.2000 10884317 PMC6772334

[B115] Newey S. E. Velamoor V. Govek E. E. Van Aelst L. (2005). Rho GTPases, dendritic structure, and mental retardation. *J. Neurobiol.* 64 58–74. 10.1002/neu.20153 15884002

[B116] Niwa R. Nagata-Ohashi K. Takeichi M. Mizuno K. Uemura T. (2002). Control of actin reorganization by slingshot, a family of phosphatases that dephosphorylate ADF/cofilin. *Cell* 108 233–246. 10.1016/S0092-8674(01)00638-9 11832213

[B117] Nobes C. D. Hall A. (1995). Rho, Rac, and Cdc42 GTPases regulate the assembly of multimolecular focal complexes associated with actin stress fibers, lamellipodia, and filopodia. *Cell* 81 53–62. 10.1016/0092-8674(95)90370-4 7536630

[B118] O’Kane E. M. Stone T. W. Morris B. J. (2003). Activation of Rho GTPases by synaptic transmission in the hippocampus. *J. Neurochem.* 87 1309–1312. 10.1046/j.1471-4159.2003.02102.x 14622110

[B119] Oh D. Han S. Seo J. Lee J. R. Choi J. Groffen J. (2010). Regulation of synaptic Rac1 activity, long-term potentiation maintenance, and learning and memory by BCR and ABR Rac GTPase-activating proteins. *J. Neurosci.* 30 14134–14144. 10.1523/JNEUROSCI.1711-10.2010 20962234 PMC5076888

[B120] Orefice L. L. Shih C. C. Xu H. Waterhouse E. G. Xu B. (2016). Control of spine maturation and pruning through proBDNF synthesized and released in dendrites. *Mol. Cell. Neurosci.* 71 66–79. 10.1016/j.mcn.2015.12.010 26705735 PMC4761458

[B121] Park J. C. Baik S. H. Han S. H. Cho H. J. Choi H. Kim H. J. (2017). Annexin A1 restores Aβ1-42-induced blood–brain barrier disruption through the inhibition of RhoA-ROCK signaling pathway. *Aging Cell* 16 149–161. 10.1111/acel.12530 27633771 PMC5242298

[B122] Parnass Z. Tashiro A. Yuste R. (2000). Analysis of spine morphological plasticity in developing hippocampal pyramidal neurons. *Hippocampus* 10 561–568. 10.1002/1098-1063(2000)10:5<561::AID-HIPO6>3.0.CO;2-X 11075826

[B123] Pchitskaya E. Bezprozvanny I. (2020). Dendritic spines shape analysis—classification or clusterization? Perspective. *Front. Synap. Neurosci.* 12:31. 10.3389/fnsyn.2020.00031 33117142 PMC7561369

[B124] Penzes P. Beeser A. Chernoff J. Schiller M. R. Eipper B. A. Mains R. E. (2003). Rapid induction of dendritic spine morphogenesis by trans-synaptic ephrinB-EphB receptor activation of the Rho-GEF kalirin. *Neuron* 37 263–274. 10.1016/S0896-6273(02)01168-6 12546821

[B125] Peters A. Kaiserman-Abramof I. R. (1970). The small pyramidal neuron of the rat cerebral cortex. The perikaryon, dendrites and spines. *Am. J. Anat.* 127 321–355. 10.1002/aja.1001270402 4985058

[B126] Pianu B. Lefort R. Thuiliere L. Tabourier E. Bartolini F. (2014). The Aβ1-42 peptide regulates microtubule stability independently of tau. *J. Cell Sci.* 127 1117–1127. 10.1242/jcs.143750 24424028

[B127] Pyronneau A. He Q. Hwang J.-Y. Porch M. Contractor A. Zukin R. S. (2017). Aberrant Rac1-cofilin signaling mediates defects in dendritic spines, synaptic function, and sensory perception in fragile X syndrome. *Sci. Signal.* 10:eaan0852. 10.1126/scisignal.aan0852 29114038 PMC5988355

[B128] Quassollo G. Wojnacki J. Salas D. A. Gastaldi L. Marzolo M. P. Conde C. (2015). A RhoA signaling pathway regulates dendritic Golgi outpost formation. *Curr. Biol.* 25 971–982. 10.1016/j.cub.2015.01.075 25802147

[B129] Rex C. S. Chen L. Y. Sharma A. Liu J. Babayan A. H. Gall C. M. (2009). Different Rho GTPase-dependent signaling pathways initiate sequential steps in the consolidation of long-term potentiation. *J. Cell Biol.* 186 85–97. 10.1083/jcb.200901084 19596849 PMC2712993

[B130] Richter M. Murtaza N. Scharrenberg R. White S. H. Johanns O. Walker S. (2019). Altered TAOK2 activity causes autism-related neurodevelopmental and cognitive abnormalities through RhoA signaling. *Mol. Psychiatry* 24 1329–1350. 10.1038/s41380-018-0025-5 29467497 PMC6756231

[B131] Ridley A. J. Hall A. (1992). The small GTP-binding protein rho regulates the assembly of focal adhesions and actin stress fibers in response to growth factors. *Cell* 70 389–399. 10.1016/0092-8674(92)90163-7 1643657

[B132] Ridley A. J. Paterson H. F. Johnston C. L. Diekmann D. Hall A. (1992). The small GTP-binding protein rac regulates growth factor-induced membrane ruffling. *Cell* 70 401–410. 10.1016/0092-8674(92)90164-8 1643658

[B133] Rong Z. Cheng B. Zhong L. Ye X. Li X. Jia L. (2020). Activation of FAK/Rac1/Cdc42-GTPase signaling ameliorates impaired microglial migration response to Aβ42 in triggering receptor expressed on myeloid cells 2 loss-of-function murine models. *FASEB J.* 34 10984–10997. 10.1096/fj.202000550RR 32613609

[B134] Rossman K. L. Der C. J. Sondek J. (2005). GEF means go: turning on Rho GTPases with guanine nucleotide-exchange factors. *Nat. Rev. Mol. Cell Biol.* 6 167–180. 10.1038/nrm1587 15688002

[B135] Rust M. B. (2015). Novel functions for ADF/cofilin in excitatory synapses – lessons from gene-targeted mice. *Commun. Integr. Biol.* 8:e1114194. 10.1080/19420889.2015.1114194 27066177 PMC4802768

[B136] Rust M. B. Gurniak C. B. Renner M. Vara H. Morando L. Görlich A. (2010). Learning, AMPA receptor mobility and synaptic plasticity depend on n-cofilin-mediated actin dynamics. *EMBO J.* 29 1889–1902. 10.1038/emboj.2010.72 20407421 PMC2885936

[B137] Sadybekov A. Tian C. Arnesano C. Katritch V. Herring B. E. (2017). An autism spectrum disorder-related de novo mutation hotspot discovered in the GEF1 domain of Trio. *Nat. Commun.* 8:601. 10.1038/s41467-017-00472-0 28928363 PMC5605661

[B138] Scott E. K. Reuter J. E. Luo L. (2003). Small GTPase Cdc42 is required for multiple aspects of dendritic morphogenesis. *J. Neurosci.* 23 3118–3123. 10.1523/jneurosci.23-08-03118.2003 12716918 PMC6742332

[B139] Shen W. Kilander M. B. C. Bridi M. S. Frei J. A. Niescier R. F. Huang S. (2020). Tomosyn regulates the small RhoA GTPase to control the dendritic stability of neurons and the surface expression of AMPA receptors. *J. Neurosci. Res.* 98 1213–1231. 10.1002/jnr.24608 32133675 PMC7216846

[B140] Shuai Y. Lu B. Hu Y. Wang L. Sun K. Zhong Y. (2010). Forgetting is regulated through Rac activity in *Drosophila*. *Cell* 140 579–589. 10.1016/j.cell.2009.12.044 20178749

[B141] Soderling S. H. Guire E. S. Kaech S. White J. Zhang F. Schutz K. (2007). A WAVE-1 and WRP signaling complex regulates spine density, synaptic plasticity, and memory. *J. Neurosci.* 27 355–365. 10.1523/JNEUROSCI.3209-06.2006 17215396 PMC3740594

[B142] Soderling S. H. Langeberg L. K. Soderling J. A. Davee S. M. Simerly R. Raber J. (2003). Loss of WAVE-1 causes sensorimotor retardation and reduced learning and memory in mice. *Proc. Natl. Acad. Sci. U.S.A.* 100 1723–1728. 10.1073/pnas.0438033100 12578964 PMC149900

[B143] Sonkar V. K. Kulkarni P. P. Dash D. (2014). Amyloid β peptide stimulates platelet activation through RhoA-dependent modulation of actomyosin organization. *FASEB J.* 28 1819–1829. 10.1096/fj.13-243691 24421399

[B144] Soosairajah J. Maiti S. Wiggan O. Sarmiere P. Moussi N. Sarcevic B. (2005). Interplay between components of a novel LIM kinase-slingshot phosphatase complex regulates cofilin. *EMBO J.* 24 473–486. 10.1038/sj.emboj.7600543 15660133 PMC548651

[B145] Sorra K. E. Harris K. M. (2000). Overview on the structure, composition, function, development, and plasticity of hippocampal dendritic spines. *Hippocampus* 10 501–511. 10.1002/1098-1063(2000)10:5<501::AID-HIPO1>3.0.CO;2-T 11075821

[B146] Stone O. J. Pankow N. Liu B. Sharma V. P. Eddy R. J. Wang H. (2019). Optogenetic control of cofilin and αTAT in living cells using Z-lock. *Nat. Chem. Biol.* 15 1183–1190. 10.1038/s41589-019-0405-4 31740825 PMC6873228

[B147] Takenawa T. Suetsugu S. (2007). The WASP-WAVE protein network: connecting the membrane to the cytoskeleton. *Nat. Rev. Mol. Cell Biol.* 8 37–48. 10.1038/nrm2069 17183359

[B148] Tang A. H. Chen H. Li T. P. Metzbower S. R. MacGillavry H. D. Blanpied T. A. (2016). A trans-synaptic nanocolumn aligns neurotransmitter release to receptors. *Nature* 536 210–214. 10.1038/nature19058 27462810 PMC5002394

[B149] Tashiro A. Yuste R. (2004). Regulation of dendritic spine motility and stability by Rac1 and Rho kinase: evidence for two forms of spine motility. *Mol. Cell. Neurosci.* 26 429–440. 10.1016/j.mcn.2004.04.001 15234347

[B150] Tashiro A. Minden A. Yuste R. (2000). Regulation of dendritic spine morphology by the Rho family of small GTPases: antagonistic roles of Rac and Rho. *Cereb. Cortex* 10 927–938. 10.1093/cercor/10.10.927 11007543

[B151] Tu G. Ying L. Ye L. Zhao J. Liu N. Li J. (2019). Dopamine D1 and D2 receptors differentially regulate Rac1 and Cdc42 signaling in the nucleus accumbens to modulate behavioral and structural plasticity after repeated methamphetamine treatment. *Biol. Psychiatry* 86 820–835. 10.1016/j.biopsych.2019.03.966 31060803

[B152] Um K. Niu S. Duman J. G. Cheng J. X. Tu Y. K. Schwechter B. (2014). Dynamic control of excitatory synapse development by a Rac1 GEF/GAP regulatory complex. *Dev. Cell* 29 701–715. 10.1016/j.devcel.2014.05.011 24960694 PMC4111230

[B153] Valdez C. M. Murphy G. G. Beg A. A. (2016). The Rac-GAP alpha2-chimaerin regulates hippocampal dendrite and spine morphogenesis. *Mol. Cell. Neurosci.* 75 14–26. 10.1016/j.mcn.2016.06.002 27297944 PMC5023278

[B154] van Galen E. J. M. Ramakers G. J. A. (2005). “Rho proteins, mental retardation and the neurobiological basis of intelligence,” in *Development, Dynamics and Pathiology of Neuronal Networks: From Molecules to Functional Circuits*, Vol. 147 eds van Pelt J. Kamermans M. Levelt C. N. van Ooyen A. Ramakers G. J. A. Roelfsema P. R. (Amsterdam: Elsevier), 295–317. 10.1016/S0079-6123(04)47022-8 15581714

[B155] Vega F. M. Ridley A. J. (2007). SnapShot: Rho family GTPases. *Cell* 29:1430. 10.1016/j.cell.2007.06.021 17604728

[B156] Verkerk A. J. M. H. Pieretti M. Sutcliffe J. S. Fu Y. H. Kuhl D. P. A. Pizzuti A. (1991). Identification of a gene (FMR-1) containing a CGG repeat coincident with a breakpoint cluster region exhibiting length variation in fragile X syndrome. *Cell* 65 905–914. 10.1016/0092-8674(91)90397-H 1710175

[B157] Wang J. Wang Y. H. Hou Y. Y. Xi T. Liu Y. Liu J. G. (2013). The small GTPase RhoA, but not Rac1, is essential for conditioned aversive memory formation through regulation of actin rearrangements in rat dorsal hippocampus. *Acta Pharmacol. Sin.* 34 811–818. 10.1038/aps.2013.3 23564082 PMC4002894

[B158] Wang P. L. Niidome T. Akaike A. Kihara T. Sugimoto H. (2009). Rac1 inhibition negatively regulates transcriptional activity of the amyloid precursor protein gene. *J. Neurosci. Res.* 87 2105–2114. 10.1002/jnr.22039 19267423

[B159] Wang Y. Du X. Wang D. Wang J. Du J. (2020). Effects of bisphenol a exposure during pregnancy and lactation on hippocampal function in newborn rats. *Int. J. Med. Sci.* 17 1751–1762. 10.7150/ijms.47300 32714078 PMC7378672

[B160] Wang Y. Zeng C. Li J. Zhou Z. Ju X. Xia S. (2018). PAK2 haploinsufficiency results in synaptic cytoskeleton impairment and autism-related behavior. *Cell Rep*. 24 2029–2041. 10.1016/j.celrep.2018.07.061 30134165

[B161] Wegner A. M. Nebhan C. A. Hu L. Majumdar D. Meier K. M. Weaver A. M. (2008). N-WASP and the Arp2/3 complex are critical regulators of actin in the development of dendritic spines and synapses. *J. Biol. Chem.* 283 15912–15920. 10.1074/jbc.M801555200 18430734 PMC2414292

[B162] Wiens K. M. Lin H. Liao D. (2005). Rac1 induces the clustering of AMPA receptors during spinogenesis. *J. Neurosci.* 25 10627–10636. 10.1523/JNEUROSCI.1947-05.2005 16291935 PMC6725855

[B163] Wu W. Du S. Shi W. Liu Y. Hu Y. Xie Z. (2019). Inhibition of Rac1-dependent forgetting alleviates memory deficits in animal models of Alzheimer’s disease. *Protein Cell* 10 745–759. 10.1007/s13238-019-0641-0 31321704 PMC6776562

[B164] Wu Y. I. Frey D. Lungu O. I. Jaehrig A. Schlichting I. Kuhlman B. (2009). A genetically-encoded photoactivatable Rac controls the motility of living cells. *Nature* 461 104–108. 10.1038/nature08241.A 19693014 PMC2766670

[B165] Xie Z. Srivastava D. P. Photowala H. Kai L. Cahill M. E. Woolfrey K. M. (2007). Kalirin-7 controls activity-dependent structural and functional plasticity of dendritic spines. *Neuron* 56 640–656. 10.1016/j.neuron.2007.10.005 18031682 PMC2118058

[B166] Yang Y. Wang X. B. Frerking M. Zhou Q. (2008). Spine expansion and stabilization associated with long-term potentiation. *J. Neurosci.* 28 5740–5751. 10.1523/JNEUROSCI.3998-07.2008 18509035 PMC2561912

[B167] Yuste R. Bonhoeffer T. (2001). Morphological changes in dendritic spines associated with long-term synaptic plasticity. *Ann. Rev. Neurosci.* 24 1071–1089. 10.1146/annurev.neuro.24.1.1071 11520928

[B168] Zamboni V. Armentano M. Saró G. Ciraolo E. Ghigo A. Germena G. (2016). Disruption of ArhGAP15 results in hyperactive Rac1, affects the architecture and function of hippocampal inhibitory neurons and causes cognitive deficits. *Sci. Rep.* 6:34877. 10.1038/srep34877 27713499 PMC5054378

[B169] Zhang H. Macara I. G. (2006). The polarity protein PAR-3 and TIAM1 cooperate in dendritic spine morphogenesis. *Nat. Cell Biol.* 8 227–237. 10.1038/ncb1368 16474385

[B170] Zhang X. Li Q. Wang L. Liu Z. J. Zhong Y. (2016). Cdc42-dependent forgetting regulates repetition effect in prolonging memory retention. *Cell Rep.* 16 817–825. 10.1016/j.celrep.2016.06.041 27396329

[B171] Zheng N. Jeyifous O. Munro C. Montgomery J. M. Green W. N. (2015). Synaptic activity regulates AMPA receptor trafficking through different recycling pathways. *ELife* 4:e06878. 10.7554/eLife.06878 25970033 PMC4451724

[B172] Zhou Q. Xiao M. Y. Nicoll R. A. (2001). Contribution of cytoskeleton to the internalization of AMPA receptors. *Proc. Natl. Acad. Sci. U.S.A.* 98 1261–1266. 10.1073/pnas.98.3.1261 11158627 PMC14742

[B173] Zhou Y. Su Y. Li B. Liu F. Ryder J. W. Wu X. (2003). Nonsteroidal anti-inflammatpry drugs can lower amyloidogenic Aβ 42 by inhibiting Rho. *Science* 302 1215–1217. 10.1126/science.1090154 14615541

[B174] Zhou Z. Hu J. Passafaro M. Xie W. Jia Z. (2011). GluA2 (GluR2) regulates metabotropic glutamate receptor-dependent long-term depression through N-cadherin-dependent and cofilin-mediated actin reorganization. *J. Neurosci.* 31 819–833. 10.1523/JNEUROSCI.3869-10.2011 21248105 PMC6632944

[B175] Zhou Z. Meng Y. Asrar S. Todorovski Z. Jia Z. (2009). A critical role of Rho-kinase ROCK2 in the regulation of spine and synaptic function. *Neuropharmacology* 56 81–89. 10.1016/j.neuropharm.2008.07.031 18718479

[B176] Zhu X. Raina A. K. Boux H. Simmons Z. L. Takeda A. Smith M. A. (2000). Activation of oncogenic pathways in degenerating neurons in Alzheimer disease. *Int. J. Dev. Neurosci.* 18 433–437. 10.1016/S0736-5748(00)00010-1 10817927

[B177] Zimering J. H. Dong Y. Fang F. Huang L. Zhang Y. Xie Z. (2016). Anesthetic sevoflurane causes Rho-dependent filopodial shortening in mouse neurons. *PLoS One* 11:e0159637. 10.1371/journal.pone.0159637 27441369 PMC4956198

